# Effect of Atmospheric-Pressure Plasma on Functional Compounds and Physiological Activities in Peanut Shells

**DOI:** 10.3390/antiox11112214

**Published:** 2022-11-09

**Authors:** Narae Han, Jinwoo Kim, Jin Hee Bae, Mihyang Kim, Jin Young Lee, Yu-Young Lee, Moon Seok Kang, Duksun Han, Sanghoo Park, Hyun-Joo Kim

**Affiliations:** 1 Department of Central Area Crop Science, National Institute of Crop Science, Rural Development Administration, Suwon 16429, Korea; 2 Department of Nuclear and Quantum Engineering, Korea Advanced Institute of Science and Technology (KAIST), Daejeon 34141, Korea; 3 Institute of Plasma Technology, Korea Institute of Fusion Energy (KFE), Gunsan 54004, Korea

**Keywords:** arachis hypogaea, by-product, plasma, functional components, physiological activity

## Abstract

Peanut (*Arachis hypogaea* L.) shell, an abundant by-product of peanut production, contains a complex combination of organic compounds, including flavonoids. Changes in the total phenolic content, flavonoid content, antioxidant capacities, and skin aging-related enzyme (tyrosinase, elastase, and collagenase)-inhibitory activities of peanut shell were investigated after treatment in pressure swing reactors under controlled gas conditions using surface dielectric barrier discharge with different plasma (NO_x_ and O_3_) and temperature (25 and 150 °C) treatments. Plasma treatment under ozone-rich conditions at 150 °C significantly affected the total phenolic (270.70 mg gallic acid equivalent (GAE)/g) and flavonoid (120.02 mg catechin equivalent (CE)/g) contents of peanut shell compared with the control (253.94 and 117.74 mg CE/g, respectively) (*p* < 0.05). In addition, with the same treatment, an increase in functional compound content clearly enhanced the antioxidant activities of components in peanut shell extracts. However, the NO_x_-rich treatment was significantly less effective than the O_3_ treatment (*p* < 0.05) in terms of the total phenolic content, flavonoid content, and antioxidant activities. Similarly, peanut shells treated in the reactor under O_3_-rich plasma conditions at 150 ℃ had higher tyrosinase, elastase, and collagenase inhibition rates (55.72%, 85.69%, and 86.43%, respectively) compared to the control (35.81%, 80.78%, and 83.53%, respectively). Our findings revealed that a reactor operated with O_3_-rich plasma-activated gas at 150 °C was better-suited for producing functional industrial materials from the by-products of peanuts.

## 1. Introduction

Peanut (*Arachis hypogaea* L.) is an important crop cultivated worldwide. Peanut pods are composed of an outer shell that usually contains two kernels. Peanut is mainly used for oil production and as food (e.g., peanut butter and roasted peanuts) [1]. The peanut shell, which is a by-product of peanut, is removed for the production of most peanut products, and accounts for about 25–30% by weight of the total peanut pod. The annual global production of peanut kernel is >44 million tons, which produces approximately 11 million tons of peanut shell [2,3]. At present, the peanut shell is considered an industrial waste product, and is used in small amounts in animal feed. Recent research has demonstrated that the peanut shell contains many bioactive and functional compounds, such as luteolin, 5,7-dihydroxychromone, and eriodictyol [4]. Various agricultural by-products are used in skin care formulations, driven by consumer demand for safe and healthy ingredients for cosmetic products [5]. As a rich source of polyphenols, and due to their biological activity, peanut shells have potential for use as an ingredient in natural cosmetics.

Conventional processing methods, including heating, roasting, and gamma and far-infrared radiation treatments, have been applied to increase the total phenolic content of peanut shells [6,7,8]. Among these methods, plasma processing has emerged as a cost-effective and green technology [9]. Plasma, as the fourth state of matter (in addition to solid, liquid, and gas), is a complex mixture of ions, electrons, excited atoms and molecules, and radicals. Recently, atmospheric-pressure (AP) plasma has been widely applied in the medicine, agriculture, and food industries [10]. Previous studies have evaluated the effect of AP plasmas under different operating conditions on the quality of food including fruit, vegetables, cereal grain, and peanuts [4,10,11]. In addition to its application to raw foods, AP plasma has been used to improve the biological activity of natural phenolic compounds in food, such as naringin, quercetin, and phlorotannin [12,13,14,15]. However, to the best of our knowledge, no studies have characterized the functional compound content and biological activity of peanut shells treated by AP plasma. Moreover, few studies have investigated the phenolic content and antioxidant activities of components in peanut shell. Therefore, this study evaluated the total phenolic content, flavonoid content, antioxidant capacity, and skin aging-related enzyme inhibitory activities of peanut shells treated in a plasma reactor.

## 2. Materials and Methods

### 2.1. Plant Materials and Reagents

The peanut cultivar “Sinpalkwang”, which is the main cultivated and harvested cultivar in Korea, was purchased from a peanut farmhouse (Gochang, Korea), washed with top water, and dried completely for 3 days at room temperature. The dried samples were manually broken to separate the peanut shells from the kernels. The separated peanut shells were used in the experiment.

All reagents and standards used in this study were purchased from Sigma-Aldrich (St. Louis, MO, USA), as were all enzymes, substrates, buffers, and positive controls for skin aging-related enzyme inhibition assays. Methanol, acetonitrile, and HPLC-grade water were purchased from J.T. Baker Inc. (Phillipsburg, NJ, USA). Nanopure water was obtained from a water purification system (Milli-Q Advantage A10; Merck Millipore, Billerica, MA, USA).

### 2.2. AP Plasma Treatment

**Figure 1:**
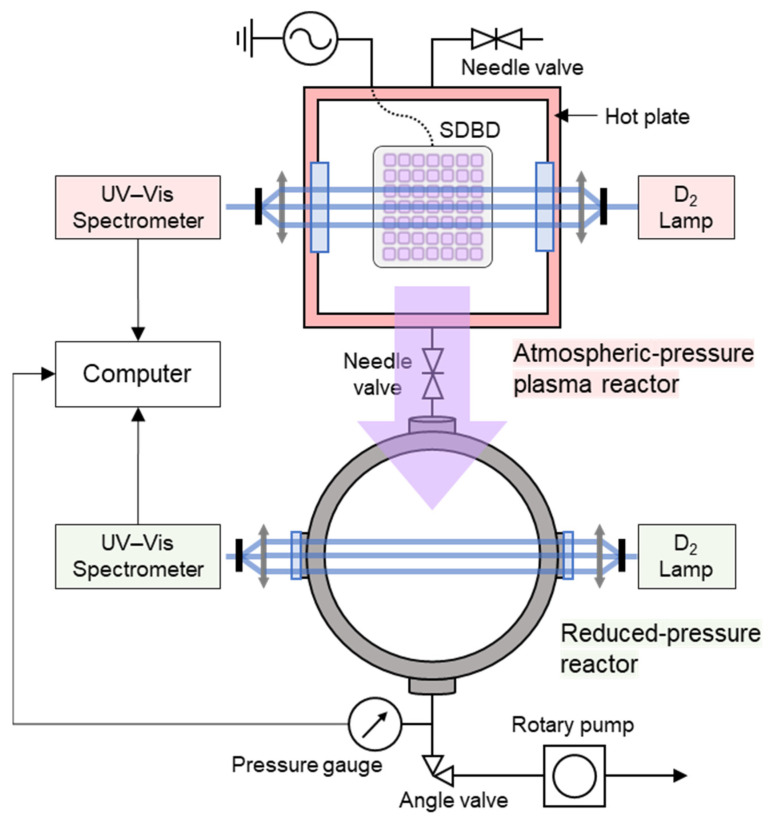
Schematics of the experimental setup for the direct and indirect plasma treatments. Samples were treated in an atmospheric-pressure plasma reactor or reduced-pressure reactor; the details of the processes are described in the main text. The O_3_ and NO_x_ concentrations in both reactors were determined using optical absorption spectroscopic systems.

Figure 1 shows the schematics of the experimental setup, which consisted of atmospheric- and reduced-pressure reactors, two identical absorption spectroscopic systems, and an electrical power system. The AP reactor was a cuboid with outer dimensions of 15 × 15 × 10 cm^3^ and an inner volume of 815 cm^3^; it was used as the plasma reactor with an internal surface dielectric barrier discharge (SDBD) source. Details of the plasma reactor can be found in our previous papers [16,17]. The SDBD source was composed of a 7 × 7 cm^2^ thin plane and grid electrodes attached to each side of a 1 mm thick, 100 × 100 mm^2^ fused silica plate. In total, 49 perforated thin film (grid) electrodes were arranged in a 7 × 7 mm^2^ rounded square pattern for surface discharge into the ambient air at the open surface of the dielectric barrier. The plane electrode was connected to an electric system consisting of a high-voltage amplifier (20/20C; Trek Inc., Medina, NY, USA) and waveform generator (33512B; Keysight, Santa Rosa, CA, USA), while the grid electrodes were grounded. A sinusoidal waveform of voltage with a frequency of 4 kHz and amplitude of 6 kV was applied to the SDBD.

As the surface plasma occupied the inner surface of the lid, reactive plasma species were produced and uniformly distributed inside the plasma reactor. One of the sample treatments was separately performed in the plasma reactor at room temperature (25 °C), and hereinafter it is referred to as O_3_ 25 °C because O_3_ was abundant. It is worth noting that it was not possible to make the plasma reactor NO_x_ dominant with the operating parameters given above. For the O_3_ 25 °C treatment, the peanut shells were placed on the bottom of the plasma reactor and treated for 45 min.

**Figure 2:**
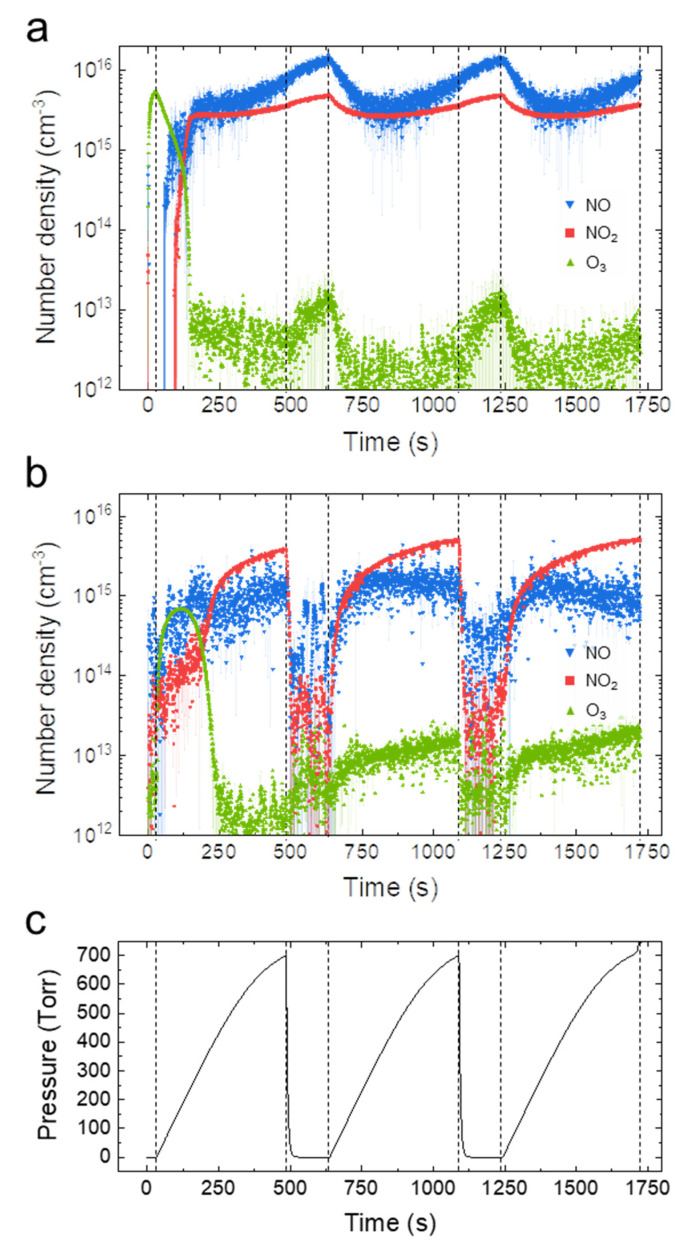
Temporal evolution of the number densities of O_3_, NO, and NO_2_ in the plasma reactor (**a**), and reduced-pressure reactor in O_3_ mode at 150 °C (**b**). Temporal variation of the gas pressure in the reduced-pressure reactor (**c**).

In this study, a novel plasma process different from the above method was designed to increase the reactivity of plasma treatments for food crops and by-products. As shown in Figure 1, an additional reactor was used in combination with a rotary pump (Uno6; Pfeiffer Vacuum, Aßlar, Germany), which was cylindrical, had an inner diameter of 15 cm, and was serially connected to the plasma reactor through a 1 m polytetrafluoroethylene tube with a needle valve. The reactor was used as a sample container; it had no internal plasma source and was periodically pressurized and depressurized in the range of 0.08–700 Torr. The novel aspects of this process were as follows. First, with the needle valves in Figure 1 fully closed, the gas pressure in the second reactor containing the sample was reduced from 760 to 0.08 Torr. The plasma reactor described above was independently operated at AP without samples to prepare the reactive oxygen and nitrogen species, including O_3_ and NO_x_. After reaching the maximum O_3_ or NO_x_ concentration in the plasma reactor, the pumping port of the second reactor was closed and the needle valves were partially opened, thereby pressurizing the second reactor. During the pressurizing phase, the highly concentrated reactive chemical species were transferred from the plasma reactor to the sample-containing second reactor by the pressure difference between the two reactors. The pressurization period was in the range of 0.08–700 Torr at 7.5 min. After pressurization, the second reactor was pumped down again while both needle valves were closed. This pressurization/depressurization process was repeated three times for each experiment. The gas pressure in the second reactor was recorded in real-time using pressure gauges (TPR280; Pfeiffer Vacuum) and a computer, as shown in Figure 2.

Following this procedure, O_3_ or NO_x_ was dominant in the plasma reactor when starting the first pressurization period of the second reactor. Under these conditions, a hot plate (MSH-20D; Daihan Scientific, Wonju, South Korea) below the plasma chamber was set at 150 °C to fix the temperature of the reactor. Thus, the treatments are hereinafter referred to as O_3_ 150 °C and NO_x_ 150 °C.

To simultaneously obtain the absolute number density of O_3_ and NO_x_ in both reactors without gas sampling, two identical ultraviolet-visible absorption spectroscopy systems were installed (one in each reactor). The absorption path lengths of the systems were 15 and 26 cm, respectively (Figure 1). The absorption spectroscopic systems comprised a deuterium lamp (DH-mini; Ocean Optics, Dunedin, FL, USA) and spectrometer (Maya2000 Pro; Ocean Optics). The lamp light was transmitted through an optical fiber (QP400-2-SR; Ocean Optics), and collimating lenses (74-UV; Ocean Optics) were used to collimate the light into the chamber and spectrometer. The interval between the two adjacent spectra in both chambers was 500 ms. All spectral data were automatically recorded by the computer, and the absolute number densities of the chemicals were calculated based on the Beer–Lambert law. The Beer–Lambert law links the intensity attenuation of optical radiation through a homogeneous sample to the density of the species present, as follows:

(1) $$I\left(\lambda \right)=I_{0}\left(\lambda \right)e^{-\sum_{i}n_{i}\sigma_{i}\left(\lambda \right)L},$$

where *I_0_(λ)* and *I(λ)* are the intensity of the incident and attenuated radiation, respectively, *n_i_* and *σ_i_* are the number density and absorption cross-section of chemical species *i*, respectively, and *L* is the absorption path length.

Special care was taken to conduct all runs with the same initial system conditions; for example, the air modified by plasma discharges during the previous experiments was substituted for ambient air. All experiments were performed using the same devices, i.e., there were no replacements. There was no aging effect on the SDBD source during the experiments.

### 2.3. Extraction Method

The AP plasma-treated peanut shells were ground into a fine powder (which passed through a 100 mesh) with a blender. The peanut shell extraction was conducted according to our previous study with slight modifications [18]. Briefly, 40 mL of 80% ethanol was added to 4 g of sample. Next, the mixture was stirred for 24 h at room temperature. The extract was centrifuged at 3000× *g* for 10 min and the supernatant was collected and evaporated in a rotary evaporator. The extract powder was re-dissolved in 100% ethanol (1:10, *w/v*) for high-performance liquid chromatography (HPLC), and in dimethyl sulfoxide (DMSO) (1:10, *w/v*) to obtain a functional compound and allow for the biological activity assay.

### 2.4. HPLC Analysis of Flavonoids in Peanut Shell Extract

The flavonoids in AP plasma-treated peanut shells were analyzed using the method applied in our previous study, with slight modifications [18]. The peanut shell extract was filtered through 0.22 µm polyvinylidene fluoride syringe filters (Acrodisc LC 13 mm syringe filter; Pall Corporation, Port Washington, NY, USA) and subjected to HPLC for flavonoid analysis (Chromaster; Hitachi Ltd., Tokyo, Japan). The flavonoids were separated using a YMC-Triart C_18_ column (150 × 3.0 mm, 5 µm particle size; YMC Europe GmbH, Dinslaken, Germany) and peak detection was performed at 280 nm with a diode array detector. The mobile phases were water with 0.1% trifluoroacetic acid (A) and acetonitrile with 0.1% trifluoroacetic acid (B) at a flow rate of 1 mL/min. The optimum gradient elution program in this study was as follows: 5% B (0–5 min), 5–25% B (5–15 min), 25–60% B (15–30 min), 60–70% B (30–37 min), 70–5% B (37–40 min), and 5% B (40–45 min). The sample injection volume was 10 µL, and the flavonoids were detected at 280 nm. The major flavonoids in the peanut shells, i.e., 5,7-dihydroxychromone, eriodictyol, and luteolin [19], were identified by comparing the individual standards with the retention times.

### 2.5. Determination of Total Phenolic and Flavonoid Contents

The AP plasma-treated peanut shells were analyzed for quantification of total phenolic and flavonoid contents according to a previous study [20]. The peanut shell extract was diluted in 1 mg/mL DMSO; the analysis was performed in triplicate.

A modified Folin–Ciocalteu procedure was used for the total phenolic content assay. Ten microliters of extract was mixed with 200 µL of 2% sodium carbonate and 10 µL of Folin–Ciocalteu reagent (diluted twice). After a 30 min incubation at 25 °C, the absorbance was read at 750 nm using an absorbance microplate reader (Elx 808; BioTek Inc., Winooski, VT, USA). The total phenolic content was expressed as the standard gallic acid equivalent (GAE) per gram of extract.

The aluminum chloride method was used for the total flavonoid content assay. Briefly, 75 µL of extract was sequentially mixed with 300 µL distilled water, 22.5 µL of sodium nitrite, 45 µL of aluminum chloride, and 150 µL of 1 M sodium hydroxide. After a 20 min incubation at 25 °C, the absorbance was read at 510 nm using an absorbance microplate reader. The total flavonoid content was expressed as the catechin equivalent (CE) per gram of extract.

### 2.6. Determination of Antioxidant Capacity

The scavenging activities of the 1,1-diphenyl-2-picrylhydrazyl (DPPH) free radical- and ferric-reducing antioxidant power (FRAP) assays were analyzed to evaluate the antioxidant activity of components in AP plasma-treated peanut shells. The assays were conducted based on the method used in our previous study, with some modifications [18]. The peanut shell extract was diluted at 1 mg/mL in DMSO and all experiments were performed in triplicate under subdued light.

#### 2.6.1. DPPH Assay

Trolox was used as the standard for the DPPH scavenging assay, and quantification was performed using standard curves. The DPPH reagent was dissolved in methanol to produce a solution concentration of 0.2 mM. Twenty microliters of extract or standard was loaded into 96-well microplates and 200 µL of DPPH solution was added. After incubation for 30 min at room temperature, the absorbance was measured at 520 nm using an absorbance microplate reader. The DPPH activity was expressed as the Trolox equivalent (TE) per gram of extract.

#### 2.6.2. FRAP Assay

Peanut shell extract (30 µL) was mixed with 180 µL of FRAP reagent and 90 µL of dH_2_O, and incubated for 10 min at 37 °C. Then, the absorbance was measured at 593 nm using an absorbance microplate reader. The FRAP reagent comprised 2.5 mL of 10 mM 2,4,6-Tris(2-pyridyl)-s-triazine in 2.5 mL of 40 mM HCl, 20 mM FeCl_3_·6H_2_O, and 25 mL of 300 mM sodium acetate buffer (pH 3.6). Iron(III) sulfate (FeSO_3_) was used as a standard in the FRAP assay.

### 2.7. Evaluation of Anti-Aging Potential

The tyrosinase, elastase, and collagenase inhibitory activities were analyzed following the enzymatic methods described by Mechqoq et al. (2022) [21] with slight modifications. The experiments were performed under subdued light in triplicate and the final DMSO concentrations did not exceed 1% of the total volume. The inhibition rate was calculated as follows:

(2) $$\mathrm{Inhibition}\;\left(\%\right)=\left(1-\frac{A_{sample}-A_{sample\;blank}}{A_{control}-A_{control\;blank}}\right)\times 100,$$

where *A_sample_*, *A_sample blank_*, *A_control_*, and *A_control blank_* are the absorbance or fluorescence of a mixture consisting of a buffer, enzyme, sample or positive control, and substrate, a mixture without enzyme (replaced with buffer), a mixture without sample (replaced with sample solvent), and a mixture without sample and enzyme, respectively.

#### 2.7.1. Anti-Tyrosinase Assay

The tyrosinase inhibitory activity of extracts was evaluated using a dopachrome method with L-3,4-dihydroxyphenylalanine (L-DOPA) as the substrate. Briefly, the samples were diluted at 1 mg/mL in 0.1 M potassium phosphate buffer (pH 6.8). Forty microliters of extract, 80 µL of buffer, and 40 µL of mushroom tyrosinase (100 units/mL) (dissolved in buffer) were mixed in 96-well microplates and incubated for 10 min at room temperature. Then, 40 µL of 10 mM L-DOPA (dissolved in buffer) was added. After a 10 min incubation, the absorbance was measured at 475 nm using an absorbance microplate reader. Kojic acid, which inhibits tyrosinase, was used as the positive control (IC_50_ = 7.11 µg/mL).

#### 2.7.2. Anti-Elastase Activity Assay

The elastase inhibitory activity of the extracts was evaluated according to the release of *p*-nitroaniline from N-succinyl-Ala-Ala-Ala-p-nitroanilide (substrate) by elastase. Prior to the assay, porcine pancreatic elastase type IV (0.45 units/mL) and substrate were dissolved in 50 mM Trizma-base buffer (pH 7.5). Peanut shell extract was diluted at 0.1 mg/mL using the same buffer. Thus, 10 µL of sample, 70 µL of Trizma-base buffer, and 5 µL of elastase were mixed in a 96-well plate. After a 10 min incubation, 20 µL of 2 mM substrate was added and the 96-well plate was incubated for 30 min. The absorbance was measured at 405 nm using an absorbance microplate reader. Elastatinal, which is a strong competitive inhibitor of elastase, was used as a positive control (IC_50_ = 0.26 µg/mL).

#### 2.7.3. Anti-Collagenase Activity Assay

The collagenase inhibitory activity of the extracts was evaluated using a spectrofluorimetric method with metalloproteinase-2 (MMP2) as the substrate. For this assay, collagenase from *Clostridium histolyticum* and MMP2 was dissolved in 50 mM Trizma-base buffer (pH 7.5) and the samples were diluted in the same buffer (1 mg/mL). In a 96-well plate, 40 µL of samples, 120 µL of Trizma-base buffer, and 40 µL of collagenase (50 µg/mL) were mixed and incubated for 10 min at 37 °C, and 40 µL of 50 mM MMP2 was then added. After a 30 min incubation at 37 °C, the fluorescence intensity was measured using an absorbance microplate reader at excitation and emission wavelengths of 320 and 405 nm, respectively. Chlorhexidine was used as a positive control in the anti-collagenase assay (IC_50_ = 25.27 µg/mL).

### 2.8. Statistical Analysis

All data are presented as the mean and standard deviation of the replicates (*n* = 3), calculated using SigmaPlot 14.0 software (Systat Software, San Jose, CA, USA). The treatments were compared using Duncan’s multiple range test at *p* < 0.05 using SPSS statistical software (version 18.0; SPSS, Inc., Chicago, IL, USA). A heatmap analysis was performed with Euclidean distance measurement, the Ward clustering algorithm, and Pearson’s correlation (between metabolites and biological activities) using MetaboAnalyst 5.0 (https://www.metaboanalyst.ca/ accessed on 15 May 2022) [22].

## 3. Results and Discussion

### 3.1. O_3_ and NO_x_ Generation in the AP Plasma Chamber

The conditions for the plasma treatments in the second reactor were determined based on the concentration and composition of reactive species produced in the plasma reactor. We focused on the chemical dynamics in the case of O_3_ at 150 °C, which produced good results (Table 1, Table 2 and Table 3). Figure 2a,b show the absolute number densities of the reactive species in the plasma reactor and second reactor, respectively. Regarding the gas pressure in the second reactor (Figure 2c), the temporal variation of the chemical densities due to pressurization and depressurization was analyzed.

As shown in Figure 2a,b, O_3_ and NO_x_ were dominant during the first cycle, while the second and third cycles were mainly governed by NO_x_ in both reactors. Interestingly, when the O_3_ produced in the plasma reactor was transferred to the second reactor, its concentration rapidly decreased, whereas NO_x_ suddenly appeared and dominated the plasma reactor. As previously demonstrated [17], once such a transition occurs, the gas-tight plasma reactor tends to stay in NO_x_ mode. The chemical transition that occurred in this work should be distinguished from similar ones previously reported at elevated gas temperatures; the detailed mechanism is not yet clear and is beyond the scope of this paper.

Assuming that there was no reaction between the gaseous chemicals and reactor surface during transport, continuity in the long-lived chemicals was considered present in both reactors. However, the NO concentration was higher than the NO_2_ concentration in the plasma reactor, with the opposite trend seen in the second reactor. Unlike for NO_x_, the O_3_ concentration in the second reactor was higher than the O_3_ concentration in the plasma reactor during the second and third periods, possibly because of the difference in the absorption rate of reactive species on the peanut shell or post-plasma reactions between reactive species during the transfer process (pressurization of the second reactor).

A spectroscopic chemical analysis was also performed for NO_x_ 150 °C, although the results are not shown here. In this case, NOx was the dominant species during the entire period in both reactors because the chemical transfer to the second reactor started after the plasma reactor was completely free of O_3_ [23].

### 3.2. HPLC Analysis of Flavonoids in AP Plasma-Treated Peanut Shell

**Figure 3:**
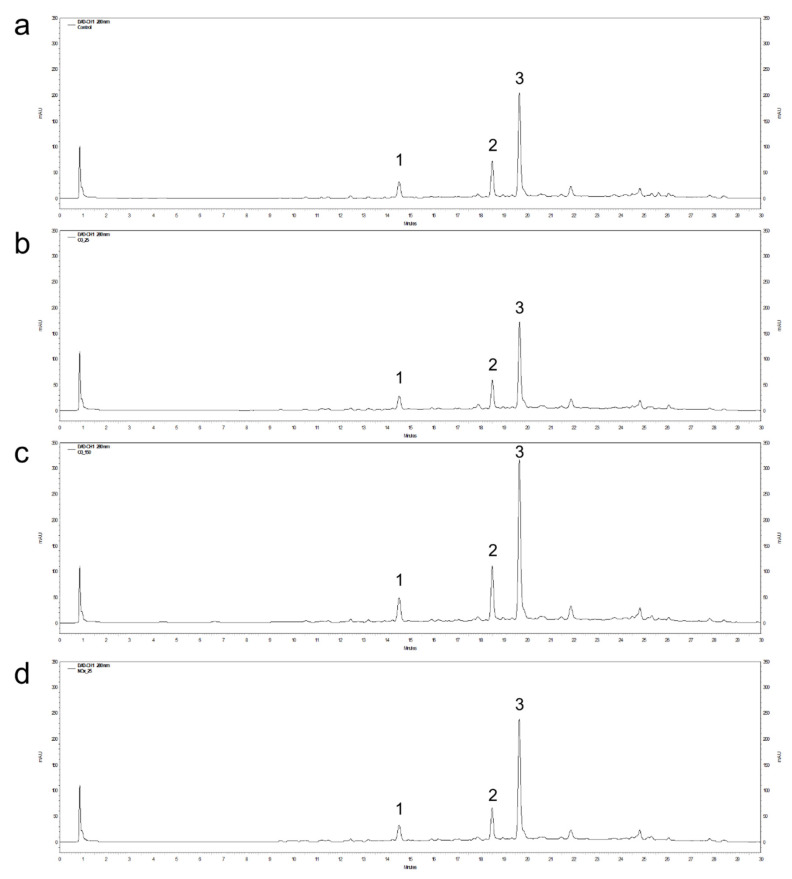
HPLC chromatograms of flavonoids from non-treated peanut shell extract (**a**) and extracts obtained by atmospheric-pressure plasma for the O_3_ 25 °C (**b**), O_3_ 150 °C (**c**), and NO_x_ 150 °C (**d**) treatments. Peak numbers 1–3 refer to 5,7-dihydroxychromone, eriodictyol, and luteolin, respectively.

The HPLC chromatograms of flavonoids and their contents from the AP plasma-treated peanut shell are shown in Figure 3 and Table 1. Three aglycon forms of flavonoid, 5,7-dihydroxychromone, eriodictyol, and luteolin, were detected and identified by comparing the individual standards with the retention times. Figure 3 shows that the composition of the extracted flavonoids obtained by the three different AP plasma treatment methods (O_3_ 25 °C, O_3_ 150 °C, and NO_x_ 150 °C) was very similar to that of the control. This result indicates that the AP plasma treatment had no effect on the composition of flavonoids, while the relative proportion of the flavonoids changed according to the radical type and/or temperature (Table 1).

**Table 1:** Content of 5,7-dihydroxychromone, eriodictyol, and luteolin (mg/g extract) in atmospheric-pressure plasma-treated peanut shells.

Treatments	5,7-Dihydroxychromone	Eriodictyol	Luteolin
Control	20.68 ± 0.74 b	30.85 ± 0.39 b	71.70 ± 0.80 c
O_3_ 25 °C	19.64 ± 0.07 b	26.51 ± 0.32 d	57.36 ± 1.00 d
O_3_ 150 °C	26.70 ± 1.08 a	40.86 ± 1.21 a	114.59 ± 0.82 a
NO_x_ 150 °C	20.59 ± 0.85 b	27.98 ± 0.45 c	83.45 ± 1.13 b

All content is presented as the mean ± standard deviation of three replicates. Different letters in the same column indicate a significant difference between treatments according to Duncan’s multiple range test at *p* < 0.05.

### 3.3. Evaluation of Functional Compound Content and Antioxidant Activity of Components in AP Plasma-Treated Peanut Shell

The changes of total phenolic and flavonoid contents in peanut shells treated by AP plasma under different radical and temperature conditions are shown in Table 2. As discussed above, the highest total flavonoid content (120.02 mg CE/g) was detected in the O_3_ 150 °C treatment. Additionally, the O_3_ 150 °C treatment significantly increased the total phenolic content in peanut shells compared with the other treatments (*p* < 0.05). The O_3_ radical-treated peanut shells had higher total phenolic and flavonoid contents, regardless of temperature, compared to the control; however, the NO_x_ radical treatment was less effective than the O_3_ radical treatment in terms of the total phenolic and flavonoid contents. In previous studies, various processing methods were applied to increase the total phenolic content of peanuts, such as heating (roasting) and gamma or far-infrared radiation treatments. These treatments may release phenolic compounds from the various cellular components in peanuts or degrade large phenolic compounds into smaller ones, resulting in an increase in the total phenolic content [6,7,8]. Zhang et al. (2014) [20] demonstrated that the ozone treatment of peanut skin increased the total flavonoid content by releasing flavonoids from their glucosidic components. In this study, it was assumed that the increase of total phenolic and flavonoid contents in peanut shells might have been caused by cell wall modification by the O_3_ radical during AP plasma exposure.

Antioxidant activity is a strong indicator of the various polyphenols in peanut, such as flavonoids, phenolic acid, isoflavones, and procyanidins [1]. The antioxidant capacity of polyphenols can be evaluated based on their free-radical scavenging activity and reductive potential. As shown in Table 2, the FRAP activity in the control peanut shell was 1.29 mM, similar to a previously reported value of 1.18 mM, while the DPPH activity in the control (156.33 mg TE/g) was lower than that reported previously (411.5 mg TE/g after correction according to mg TE/g) [18]. The variation in antioxidant activity of components in peanut shells reported in the literature is probably due to differences in harvest times and extraction methods. The DPPH and FRAP activities of peanut shell extracts were significantly affected by the AP plasma treatment (*p* < 0.05), and the activities were in the following order (highest first): O_3_ 150 °C, O_3_ 25 °C, NO_x_ 150 °C, and control. In this study, the DPPH and FRAP activities of AP plasma-treated peanut shells were strongly correlated with the total phenolic content (*r_DPPH_* = 0.697 and *r_PFRAP_* = 0.792) and flavonoid content (*r_DPPH_* = 0.681 and *r_PFRAP_* = 0.862) at *p* < 0.05. Therefore, it was assumed that AP plasma exposure combined with O_3_ 150 °C improved the antioxidant activities of the components in peanut shell by increasing the total phenolic and flavonoid contents.

**Table 2:** Content of functional compounds and antioxidant activities of components in atmospheric-pressure plasma-treated peanut shells.

Treatments	TPC ^(1)^ (mg GAE/g)	TFC (mg CE/g)	DPPH (mg TE/g)	FRAP (mM/g)
Control	253.94 ± 4.19 b	111.74 ± 1.50 c	156.33 ± 4.34 c	1.29 ± 0.00 c
O_3_ 25 °C	262.76 ± 1.32 ab	115.83 ± 0.30 b	174.50 ± 1.64 b	1.38 ± 0.03 b
O_3_ 150 °C	270.70 ± 5.70 a	120.02 ± 1.36 a	181.30 ± 1.38 a	1.46 ± 0.02 a
NO_x_ 150 °C	254.72 ± 6.29 b	109.05 ± 1.68 d	170.41 ± 1.97 b	1.32 ± 0.02 c

All content is presented as the mean ± standard deviation of three replicates. Different letters in the same column indicate a significant difference between treatments according to Duncan’s multiple range test at *p* < 0.05. ^(1)^ TPC, total phenolic compound content; GAE; gallic acid equivalent; TFC, total flavonoid content; CE; catechin equivalent; DPPH, 1,1-diphenyl-2-picrylhydrazyl; TE, Trolox equivalent; FRAP, ferric-reducing antioxidant power.

### 3.4. Evaluation of Skin Aging-Related Enzyme Inhibitory Activities in AP Plasma-Treated Peanut Shells

This study focused on the anti-aging properties of AP plasma-treated peanut shells because many studies have shown that peanuts are a good source of antioxidants [1]. To investigate the anti-aging properties, enzyme inhibition activities against tyrosinase, elastase, and collagenase were tested; the results are presented in Table 3.

Tyrosinase catalyzes the hydroxylation of L-tyrosine to L-DOPA and the oxidation of L-DOPA to dopaquinone, and these reactions are considered part of a melanin biosynthetic pathway [21]. Melanin plays an important role in protecting the skin from ultraviolet rays; however, excessive melanin accumulation leads to hyperpigmentation disorders, such as melisma, freckles, and age spots [24]. The development of tyrosinase inhibitors for use in cosmetics is therefore important for the control of melanin hyperpigmentation and skin whitening. The tyrosinase inhibitory activity in peanut shell extract is significantly increased by AP plasma treatment, regardless of the radicals or temperature, compared to the control (35.81%) (*p* < 0.05). In this study, tyrosinase inhibition was greatest (55.72%) in the O_3_ 150 °C treatment, followed by NO_x_ 150 °C and O_3_ 25 °C.

Elastin and collagen, an extracellular matrix protein, are responsible for the elasticity, strength, flexibility, and resiliency of skin. Elastase and collagenase degrade the elastin fibers and collagen network, respectively, and the activation of these enzymes results in a decrease in the elasticity of skin, the appearance of wrinkles, and skin aging [21]. In this study, AP plasma treated with the O_3_ radical showed enhanced inhibition of elastase and collagenase (Table 3). Similar to the tyrosinase inhibition assay, the most potent anti-elastase and anti-collagenase effects were observed for the O_3_ 150 °C treatment (85.69% and 86.43%, respectively). In contrast, the inhibition rate of the AP plasma with NO_x_ radical treatment was slightly lower than that of the control.

As with the antioxidant activity mentioned above, these skin aging-related enzyme inhibitory effects of AP plasma treatments are probably due to the polyphenols in peanut shell extract. In previous studies, many anti-aging products were reported in green tea, grape seed, coconut, and argan extracts [21,25]; however, the skin aging-related enzyme inhibitory activities of peanut shell have not yet been elucidated. Recently, many cosmetic companies have shown an interest in natural substances that have anti-tyrosinase, anti-elastase, and anti-collagenase activities. Thus, our results will be of commercial interest; the application of AP plasma to peanut by-products could produce materials for the cosmetic industry.

**Figure 4:**
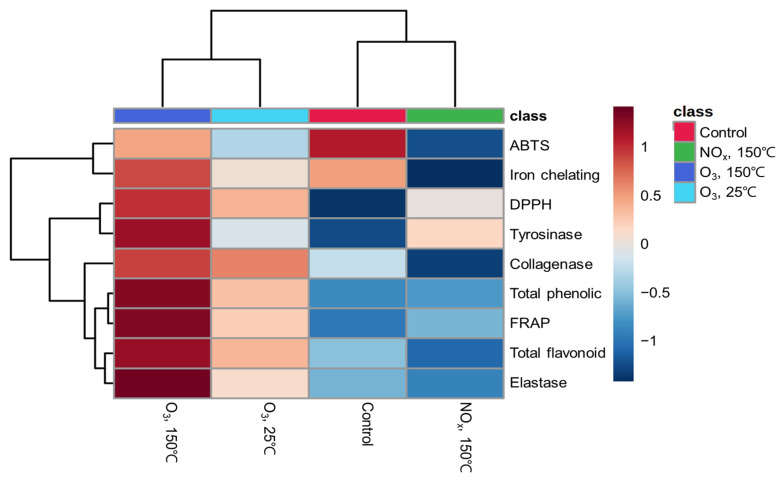
Normalized response of the functional compounds and physiological activities of peanut shells according to the atmospheric-pressure plasma treatment.

The results of our study are summarized in the normalized heatmap graph in Figure 4. The O_3_ 150 °C treatment produced the highest concentration of functional compounds and highest biological activity. Based on our findings, the potent tyrosinase, elastase, and collagenase inhibitory activities of AP plasma with O_3_ radicals at 150 °C could be involved in the high antioxidant potential of peanut shells and presence of large amounts of phenolic compounds and flavonoids.

**Table 3:** Skin aging-related enzyme inhibitory effect (%) in atmospheric-pressure plasma-treated peanut shells.

Treatments	Tyrosinase	Elastase	Collagenase
Control	35.81 ± 3.44 c	80.78 ± 1.69 bc	83.53 ± 2.47 ab
O_3_ 25 °C	44.25 ± 5.24 b	82.49 ± 0.16 b	85.72 ± 3.20 a
O_3_ 150 °C	55.72 ± 4.16 a	85.69 ± 0.16 a	86.43 ± 1.21 a
NO_x_ 150 °C	46.39 ± 3.57 b	80.01 ± 1.70 c	80.76 ± 1.40 b

Extracts were tested at 1 mg/mL for tyrosinase and collagenase inhibition assay and 0.1 mg/mL for elastase inhibition assay. The values are presented as the mean ± standard deviation of three replicates. Different letters in the same column indicate a significant difference between treatments according to Duncan’s multiple range test at *p* < 0.05.

## 4. Conclusions

Our pressure swing reactor controlled by plasma reactive gases will enable peanut shell to serve as a valuable resource. This is the first study to extensively evaluate the functional compounds and biological activities of peanut shells treated in a plasma reactor under different conditions, i.e., O_3_ (25 and 150 °C) and NO_x_ (150 °C). The O_3_ 150 °C treatment improved the antioxidant activities and skin aging-related enzyme inhibitory effect of peanut shells by increasing the total phenolic and flavonoid contents. Therefore, we conclude that the application of AP plasma combined with O_3_ radicals at 150 °C is an efficient method for treating peanut shells prior to their use in industrial applications.

## Data Availability

Not applicable.

## Abbreviations

AP, atmospheric-pressure; CE, catechin equivalent; DMSO, dimethyl sulfoxide; DPPH, 1,1-diphenyl-2-picrylhydrazyl; FRAP, ferric-reducing antioxidant power; GAE, gallic acid equivalent; HPLC, high-performance liquid chromatography; L-DOPA, L-3,4-dihydroxyphenylalanine; MMP2, metalloproteinase-2; SDBD, surface dielectric barrier discharge; TE, Trolox equivalent.
